# Disrupted modular and hub topology in right temporal lobe epilepsy: a multimodal MRI network analysis

**DOI:** 10.3389/fneur.2025.1618388

**Published:** 2026-01-12

**Authors:** Chuanyong Qu, Jinou Zheng, Zexiang Chen, Cuimi Luo, Dongying Huang, Ligen Fan

**Affiliations:** 1 People's Hospital of Ningxia Hui Autonomous Region, NingXia Medical University, Yinchuan, China; 2 First Affiliated Hospital, Guangxi Medical University, Nanning, China

**Keywords:** right temporal lobe epilepsy, multimodal MRI, brain network, functional connectivity, neuroimaging, topology

## Abstract

Right temporal lobe epilepsy (rTLE) is associated with disruptions in functional brain networks and structural connectivity, yet underlying mechanisms remain unclear. This study investigated the alterations in modular interactions, connector hub (CH) topology, and related structural changes in rTLE patients. It included 30 rTLE patients and 30 matched healthy controls (HCs), all of whom underwent resting-state functional MRI (rs-fMRI), diffusion-weighted imaging (DWI), and volumetric MRI (vMRI). Functional networks were analyzed by assessing modular interactions, functional connectivity (FC), and CH topological properties. White matter microstructural differences were examined using tract-based spatial statistics (TBSS), while cortical morphometry was evaluated in key CH regions. Compared with HCs, rTLE patients showed reduced modularity (Q), small-world index (*σ*), and clustering coefficient (*γ*), along with enhanced modular interactions, particularly between the supplementary motor area (SMA) and inferior temporal gyrus (ITG). CHs exhibited increased participation coefficient (PC), within-module degree z-score (WMD), and local efficiency. Structural analyses revealed reduced fractional anisotropy (FA) and increased radial diffusivity (RD) in the corpus callosum, as well as cortical thinning in the ITG and SMA. We confirmed that rTLE is characterized by disrupted modular architecture and CH topology, leading to network reorganization and associated structural abnormalities. These findings offer new insights into rTLE pathophysiology.

## 1 Introduction

Temporal lobe epilepsy (TLE) is the most prevalent focal epilepsy in adults, frequently exhibits drug resistance along with a propensity for secondary generalized seizures (1). Advances in invasive electroencephalography (EEG) and insights gained from post-surgical recurrence have revealed that the cerebral substrates underlying TLE are highly complex. These substrates not only encompass various regions within the temporal lobe but also involve extratemporal networks, which can significantly influence the extent of the epileptogenic zone and consequently impact seizure outcomes (2). In TLE, epileptic foci can simultaneously involve both the intrinsic structures of the temporal lobe and interconnected structures in neighboring regions. This network concept elucidates how a spatially confined focal abnormality can affect the entire brain, leading to diverse clinical manifestations. The brain network disorder associated with TLE is considered a result of altered spatiotemporal organization within the temporal lobe and subsequent changes in additional brain structures (3).

The modular structure is a critical characteristic of brain networks, in which the nodes within the same module exhibit stronger connections among themselves compared to those between different modules (4). This modular architecture can limit the spread of lesions to other brain regions, thereby reducing the likelihood of widespread effects (5). Modeling studies have demonstrated that modularity breakdown increases the tendency to hyper-synchronized and seizure-like dynamics, which may play a key role in the propagation of generalized seizures. Typically, within-module degree (WMD) and participation coefficient (PC) are utilized to quantify each node’s connectivity within its own module relative to other modules. Previous studies have shown that TLE patients exhibit significant functional network imbalances, characterized by increased PC and decreased WMD in cortico–cortical networks, while these metrics are reversed in thalamocortical networks (6, 7). In addition, nodes with high PC and high WMD are regarded as connector hubs (CHs) (8), which are thought to coordinate connectivity and integrate information across modules according to task demands (9). However, alterations in the topological properties of CHs within the functional brain networks of TLE, and their subsequent influence on inter-modular interactions, remain underexplored and warrant further investigation.

While functional connectivity is shaped and constrained by underlying anatomical structure, the relationship between function and structure remains unclear (10). Investigations into anatomical structure in epilepsy offer valuable insights into the extensive alterations in gray matter morphology and white matter connections that are associated with functional connectivity and various clinical syndromes. Structural and functional connectivity are more tightly coupled when functional connectivity shows more integrated network topology, and direct structural connections mediate a considerable proportion of informational exchange within the functional modules (11). Structural networks facilitate the segregation and integration of information through network modules and hubs (12), where segregation is influenced by the strength of functional connectivity within and between modules, and integration is determined by the strength of all functional connections mediated by network hubs. Furthermore, the widespread gray matter alterations in temporal lobe epilepsy (TLE) are of equal importance. In unilateral TLE patients, bilaterally distributed cortical thinning—accompanied by superficial white matter changes and reduced functional connectivity,—is closely associated with verbal memory impairment (13).

MRI has provided measures to non-invasively detect lesions causing epilepsy. Advanced multimodal MRI techniques—such as resting-state fMRI (rs-fMRI), diffusion-weighted imaging (DWI), and volumetric MRI (vMRI)—facilitate the investigation of both functional and structural connectivity in the brain, particularly in relation to the anatomical alterations observed in temporal lobe epilepsy (TLE) (14–17). Network analyses further enhance our understanding of whole-brain anomalies associated with focal TLE. Extensive functional network alterations in TLE have been widely confirmed, including changes of small-world topology, increased clustering coefficient, altered distribution of network hubs, and less segregated network organization (18, 19), while structural connectivity analyses show widespread decreased fractional anisotropy (FA) in white matter fiber bundles (20). Additionally, vMRI analyses have demonstrated not only widespread bilateral neocortical atrophy in TLE (21), but also involvement of subcortical structures such as the thalamus (22), which are associated with the altered structural connections (SCs) between them (23).

Although previous studies have documented the widespread impairments in brain anatomical structure and both functional and structural connectivity in temporal lobe epilepsy (TLE), no systematic investigation has examined the relationship between information interactions among TLE functional brain network modules and changes in the topological properties of central hubs (CHs) associated with alterations in underlying anatomical structure. In this study, we employed three distinct MRI modalities—resting-state functional MRI (rs-fMRI), diffusion-weighted imaging (DWI), and volumetric MRI (vMRI)—to evaluate modular interactions, the topological properties of CHs, and underlying structural changes, along with the structural connectivity and anatomical structure, in patients with right temporal lobe epilepsy (rTLE). These findings were compared with those from healthy controls to systematically explore potential neuropathophysiological mechanisms in TLE patients.

## 2 Methods and materials

### 2.1 Participants

Thirty patients with right temporal lobe epilepsy were recruited from the First Affiliated Hospital of Guangxi Medical University epilepsy center between the years 2018 and 2020. Comprehensive clinical assessments, along with detailed neurological history and physical examination, EEG, MRI, and neuropsychological evaluation were performed by two neurologists, specialized in epilepsy, to confirm the diagnosis according to the International League Against Epilepsy (ILAE) diagnosis and classification criteria (24, 25). The inclusion criteria for rTLE patients were as follows: (1) Clinical seizure symptoms were consistent with origin in the right temporal lobe. (2) Electroencephalography (EEG) showed that epilepsy originated from the right temporal lobe. (3) There was no identifiable lesion in the brain MRI, except for right hippocampal sclerosis. (4) All patients have been taking antiepileptic drugs (ASM) regularly. All rTLE patients experienced ≥1 bilateral tonic–clonic seizure. When ictal EEG/video-EEG documented a focal onset with subsequent bilateral tonic–clonic evolution, events were classified as focal-to-bilateral tonic–clonic seizures (FBTCS). When the onset was not captured and focal onset could not be established, events were classified as tonic–clonic seizures of unknown onset. No patient was labeled as having a generalized-onset tonic–clonic seizure. The exclusion criteria were as follows: (1) the presence of other neurological disorders or diseases in other systems; (2) evidence of secondary damage likely to cause seizure; (3) diagnosis of multifocal epilepsy or extra-rTLE; (4) abnormal structural findings on MRI, except for right hippocampal sclerosis; (5) the presence of depression, anxiety, dementia or severe mental disorders (26, 27); (6) inability to cooperate with the examination.

Thirty healthy controls (HCs) were also recruited. All healthy subjects had no history of neurological disorders or any disease in other systems. Detailed demographic and clinical characteristics of all subjects are presented in Table 1. The study was approved by the Medical Research Ethics Committee of the First Affiliated Hospital of Guangxi Medical University. Each participant provided written informed consent. All procedures performed in this study adhered to the principles outlined in the Declaration of Helsinki.

**Table 1:** Demographic and clinical characteristics of all subjects.

Variables	rTLE (*n* = 30)	HCs (*n* = 30)	*P*-value
Sex (male/female)	11/19	14/16	0.43^a^
Age (years)	31.10 ± 7.70	29.00 ± 7.93	0.30^b^
Age of onset (years)	20.14 ± 9.20	–	–
Duration of disease (years)	11.00 ± 6.25	–	–
Duration of ASM therapy (years)	9.40 ± 6.43	–	–
Monotherapy/polytherapy	14/16	–	–
With/without HS in MRI	10/20	–	–

Data were represented as mean ± standard deviation (SD), except for “Sex.” For all tests, *p* < 0.05 was considered statistically significant. rTLE, right temporal lobe epilepsy; HCs, health controls; ASM, antiepileptic drugs; HS, hippocampal sclerosis.

a *p* value was calculated using the Chi-square test.

b *p* value was calculated using a two-sample *t*-test.

### 2.2 MRI data acquisition

A 3.0 T scanner (Philips, Eindhoven, and Netherlands) was used to acquire rs-fMRI, DWI, and high-resolution T1-weighted anatomic imaging data from each subject. MRI were acquired in axial slices. Rs-fMRI data were acquired using an echo-planar imaging sequence (repetition time/echo time (TR/TE) = 2,000/30 ms); flip angle = 90°; field of view (FOV) = 220 mm × 220 mm; slice number = 41; matrix size = 64 × 64; voxel size = 3.44 mm × 3.44 mm × 4 mm; time points = 225 (53 subjects, 30 HC, 23 rTLE), or slice number = 31; time points = 180 volumes (7 subjects, all rTLE); matrix size = 64 × 64; voxel size = 3.44 mm × 3.44 mm × 6 mm; acquisition time = 7min30s for 225 volumes and 6 min for 180 volumes, respectively. DWI data were acquired using a 33-directional diffusion weighted sequence scan with a b-value of 1,000 s/mm^2^ and an additional b = 0 volume (TR/TE = 12,000/68 ms; flip angle = 90°; FOV = 224 mm × 224 mm; slice thickness = 2.0 mm; matrix size = 112 × 112; voxel size = 2 mm × 2 mm × 2 mm); acquisition time = 14 min 16 s. T1-weighted data were acquired using a 3D turbo field echo sequence (TR/TE = 7.8/3.46 ms; flip angle = 9°; FOV = 256 mm × 256 mm; slice thickness = 1 mm; matrix size = 256 × 256; voxel size = 1 mm × 1 mm × 1 mm); acquisition time = 5 min 50 s. The total acquisition time = 27 min 36 s or 26 min 06 s. During the MRI acquisition, all participants were supine in the scanner with their heads comfortably fixed with padding, and with a headset to reduce noise. They were instructed to stay awake with their eyes closed, avoid thinking about anything in particular, and remain motionless.

### 2.3 Rs-fMRI imaging preprocessing and functional network construction

Preprocessing of functional imaging datasets was performed using DPABI toolbox,^1^ which runs on MATLAB R2018b (The Math Works, Natick, Massachusetts, USA). The rs-fMRI images were processed by: (1) removing the first 10 volumes to ensure magnetization equilibration; (2) correcting the slice timing and realigning the remaining images to the mean volume to correct for head movements; (3) All T1-weighted images served as high-resolution anatomical references during the DARTEL-based normalization of rs-fMRI images to the MNI template, ensuring precise spatial alignment; moreover, comprehensive visual quality assessments were systematically performed using the DPABI toolbox’s built-in quality control module, which included slice-by-slice inspection of normalization accuracy, and motion artifact detection. This process confirmed that all datasets exhibited excellent registration fidelity and met our stringent head-motion thresholds (translation <3 mm, rotation <3°), with no exclusions required due to suboptimal data quality; (4) resampling the images to a voxel size of 3 × 3 × 3 mm^3^ and spatially smoothing the normalized functional data with a 4 mm full width at half maximum (FWHM) Gaussian kernel; (5) baseline correction excluding linear and quadratic trends; (6) regression of potential confounding signals of head motion parameters, the averaged white matter signal, and the cerebrospinal fluid signal; and (7) application of a 0.01–0.08 Hz band-pass filter to minimize influences of low-frequency drift and high-frequency physiological noise. No subject exhibited head motion greater than 3 mm of translation and/or >3 degrees of rotation.

The DPABI toolbox was also used to set up the functional networks comprising nodes and edges. Each node represents an individual brain region, and each edge represents the FC between two nodes. The nodes were defined according to the automated anatomical labeling atlas (28) (AAL90) which contains 90 brain regions of interest (ROIs), with cerebellum and brain stem removed, and edges were defined as the Pearson correlation coefficients of the functional time series between all pairs of ROIs, where were Fisher z-transformed and normalized to zero-mean and unit variance. Next, edges with negative FC values were set to 0, keeping only the positive values. Finally, an undirected weighted positive resting-state FC matrix (90 × 90) for every participant was constructed.

### 2.4 Community partitions of functional brain network

To ensure the stability and accuracy of the community partitions, and to facilitate statistical comparison between the two groups, we used a data-driven multi-iterative generalization of the Louvain community detection algorithm to construct a community partitions template at the overall level (29–31). Using the MATLAB Brain Connectivity Toolbox (BCT^2^), the main steps were processed as follows: (1) The same sparsity was set for each subject’s FC matrix to threshold and construct an undirected weighted network ensuring that each subject’s connection network had the same number of nodes and edges to facilitate statistical comparison. (2) The Louvain community algorithm was run 1,000 times on the thresholding matrix of each subject, yielding 1,000 partitions. (3) The probability that each network node in a single participant belongs to the same community was computed in 1,000 community partitions, and a consensus classification matrix, D, was constructed at the individual level; (4) a threshold, *τ* = 0.2, was applied to the matrix D, such that elements D_ij_ with a value greater than τ were retained, and values less than τ were set to zero. (5) The Louvain community algorithm was run again on this thresholding matrix D 1,000 times, yielding another 1,000 partitions. (6) Steps (3)–(5) were repeated until the consensus matrix had a block-diagonal structure in which all edge weights equaled one for node pairs in the same community and zero otherwise. Finally, a stable group community partitions template at the overall level was formed.

### 2.5 Modularity Q and the modular interaction

The overall group community partitions template obtained above was applied to each subject, and the modularity Q of each subject was calculated under the sparsity range of 5–30% (step size 1%). The area under the curve (AUC) of the Q value was then calculated in this sparsity range. Finally, a two-sample *t*-test was used to compare AUC differences between the two groups under the sparsity range and the Q value under 15% sparsity. In this study, we defined the average strength of modular interaction (ASMI) as the ratio of the sum of the strengths of all connecting edges between two modules divided by the number of their connecting edges. The numerical calculation was performed with the GRETNA toolbox,^3^ as also runs in MATLAB. To facilitate statistical analysis, we choose a 15% sparsity threshold, which is commonly used in the literature (6, 32, 33), to compare the pairwise modular interactions between the two groups. Because the two sets of ASMI data did not confirm to normal distribution and/or homogeneity of variance, a permutation test (5,000 times, two-tailed) was used, and FDR correction was performed.

### 2.6 Selection and analysis of CHs in functional network modules

The information exchange between network modules greatly depends on the information transmission of CHs within the module. As mentioned before, PC and WMD can be used to accurately quantify the extent to which a node participates in information exchange inside and outside the module. PC is used to measure the diversity of node cross-module connections, and WMD is used to measure the connectivity to other nodes in the same module. Therefore, we used the PC/WMD ratio of a CH node to quantify the proportion of the node’s contribution to the internal and external communication of the module. An increase in the PC/WMD ratio indicates that between module communication is enhanced, and conversely, a reduced PC/WMD ratio indicates the enhancement of information exchange within the module. To clarify the differences in the number of CHs and information dissemination attributes of the functional networks, we calculated the PC and WMD of each node in the individual network at a sparsity of 15% and then divided the nodes according to PC and WMD values. There is currently no established standard or method for dividing CHs (8, 34, 35), and smaller networks may have a restricted range of measures, making it difficult to draw distinctions between all the topological roles of each node. Thus, according to our FC network size and based upon PC and WMD network metrics, we defined CHs in the network as nodes that had PC > 0.3 and WMD > mean + SD of WMD (36, 37). We compared the total number and the average CH PC/WMD ratio between the two groups to explore changes in the number and attributes of CHs in the brain functional connection network of rTLE patients.

### 2.7 Analysis of FC networks constructed by major CHs

Global differences in the major CHs’ FC network components between rTLE patients and HCs were examined with network-based statistics (NBS) (38) using the NBS toolbox^4^ and following these sequences: (1) Each of the 90 functional network nodes designated as a CH in all subjects was counted and sorted in descending order, then the top 25% (i.e., the nodes defined as the CHs in the most subjects, a total of 22, Table 2) were selected as ROIs to build a 22*22 major CHs’ FC matrix for each subject. (2) Mean functional connectivity strength changes were calculated with permutation tests (5,000 times) between two groups as in connection-wise analysis. (3) Network components of interconnected edges with an uncorrected *p*-value < 0.005 were retained. (4) The network components with a *p*-value < 0.025 (one-tailed) were considered statistically significant.

**Table 2:** The top 22 ROIs (defined as CH in the most subjects).

ROI (Label name in AAL2 template)	Defined as CH in how many subjects	ROI (Label name in AAL2 template)	Defined as CH in how many subjects
Temporal_Sup_R	49	Supp_Motor_Area_R	15
Temporal_Sup_L	48	Precuneus_L	15
ParaHippocampal_R	32	Temporal_Pole_Sup_L	15
Frontal_Sup_R	31	Frontal_Sup_Medial_L	14
Temporal_Mid_L	24	Calcarine_L	14
Temporal_Inf_R	23	Temporal_Inf_L	14
Temporal_Mid_R	21	Cuneus_R	13
Lingual_R	18	Frontal_Mid_L	12
Occipital_Mid_L	18	Supp_Motor_Area_L	12
Temporal_Pole_Sup_R	18	Thalamus_L	12
Frontal_Sup_L	15	SupraMarginal_L	11

### 2.8 Topological analysis of FC networks and CHs

The GRETNA toolbox was used to calculate global and regional FC network metrics. The small-world attributes of each subject’s FC network including normalized clustering coefficient (Gamma, *γ*), normalized characteristic shortest path length (Lambda, *λ*), and small-worldness index (Sigma, *σ* = γ/λ), and the clustering coefficient, local efficiency, and degree centrality of the CH in the CHs showing group differences were calculated separately for 5–30% sparsity (step size 1%). Then, these topological metrics were standardized by the corresponding metrics generated from 1,000 random networks which preserve the same number of nodes and edges, and the same degree distribution. Finally, these topological metrics were used to calculate the AUC over the sparsity ranges. Permutation tests (5,000 times, two-tailed) were used for those data.

### 2.9 Structural interhemispheric brain connectivity analysis

DWI data were preprocessed and analyzed with the PANDA toolbox^5^ based on FMRIB Software Library (FSL^6^) and run in MATLAB. Smith and colleagues proposed a tract-based spatial statistics (TBSS) (39) approach that performs statistical analysis along individual white matter skeletons, thereby reducing co-registration misalignment across subjects and enhancing the localization of abnormalities. TBSS was performed to identify the main white fiber skeleton abnormalities across the whole brain, with the following processing steps: (1) Individual diffusion metrics (FA, MD, AD, and RD) were calculated after standard preprocessing including motion correction, eddy current and geometric distortions, brain extraction. (2) Each individual image was registered to the standard target template using nonlinear registration. (3) All registered FA images were used to generate the average FA template, and then the skeleton was extracted (thresholded at a mean FA value of 0.2). (4) The mean FA skeleton was used as a mask, and 5,000 permutations were run to compare the diffusion metrics between two groups. The significance threshold for between-group differences was set at *p* < 0.05 (two-tailed, family-wise error rate (FWE) corrected for multiple comparisons) using the threshold-free cluster enhancement (TFCE) option in the FSL “randomize” toolbox.

### 2.10 Anatomical structure measurement of CHs

FreeSurfer (version 6.0^7^) was used to calculate the anatomical structure metrics (cortical thickness, cortical volume, and surface area) of the CHs showing differential between group network components. This method uses a standard auto-reconstruction algorithm to reconstruct the cortical surface, including motion correction, tissue intensity inhomogeneity normalization, brain extraction, transformation to Talairach-like space, segmentation of gray/white matter, automated topology correction, and surface deformation. Quality control of the output was carefully inspected by two trained investigators who were blinded to the subjects’ classification. Finally, each image was resampled onto the FreeSurfer average subject (fsaverage) and smoothed with a 15-mm full-width at half-maximum (FWHM) surface-based Gaussian kernel.

The CH cortical mask was made as follows: First, the AAL90 template was mapped from the MNI152 standard space to the FreeSurfer fsaverage space to complete the template conversion from voxel-based to cortical surface-based, and then the CH region was extracted in the surface-based cortical template as a mask.

Anatomical structure metrics were compared between groups using FreeSurfer’s general linear model. A Monte Carlo method (with 5,000 iterations per simulation) was used for the multiple comparison correction. The significance level was set at a vertex-wise *p*-value < 0.001 (uncorrected) and a cluster-wise *p*-value < 0.05 (two-tailed and corrected for multiple comparisons).

### 2.11 Quality control

For imaging quality control (visual + quantitative), we used DPABI’s built-in QC module for slice-by-slice inspection of normalization accuracy and motion-artifact screening (visual inspection). We also enforced *a priori* motion thresholds (translation <3 mm; rotation <3°); no subjects exceeded these limits or were excluded. In preprocessing, we additionally regressed head-motion parameters, WM, and CSF signals and applied a 0.01–0.08 Hz band-pass filter. For graph construction and community detection (robustness), community structure was evaluated over a wide sparsity range (0.05–0.3, step = 0.01), and global metrics were summarized as AUC; figure captions note 95% CIs from 1,000 bootstrap iterations, illustrating stability across sparsity. For modular partitions, we used a multi-iterative consensus Louvain approach (1,000 runs per subject, consensus matrix threshold *τ* = 0.20, repeat until block-diagonal convergence), which reduces the dependency on any single run/parameter choice. For functional-network metrics statistical inference, we used permutation tests (5,000, two-tailed) and applied FDR where indicated; node- and component-level results in tables/figures mark permutation/FDR significance explicitly. We also standardized topological metrics against 1,000 degree-preserving random networks before AUC computation.

## 3 Results

### 3.1 Demographic characteristics

No significant differences in age or sex were observed between the rTLE patients and healthy controls (HCs) (Tables 1, 3).

**Table 3:** Demographic and clinical data of the patients with rTLE.

Patient	Sex	Age (years)	Duration of disease (years)	Seizure type	Interictal EEG discharge	ASMs currently used	Duration of ASM therapy (years)	MRI
1	F	46	7	FBTCS	Right AT	LTG + LEV	6	–
2	F	23	21	GTCS	Right AT	OXC + VPA	19	rHS
3	F	41	6	GTCS	Right AT	OXC	4	–
4	M	29	15	FBTCS	Right MT	LTG + CBZ	13	–
5	F	23	12	GTCS	Right AT and MT	LTG + VPA	11	rHS
6	M	32	7	GTCS	Right AT	LAC + VPA	5	rHS
7	M	31	20	GTCS	Right MT	LTG + VPA	18	rHS
8	F	24	6	FBTCS	Right MT	LTG	5	–
9	F	25	10	GTCS	Right MT	LTG	8	–
10	F	36	4	FBTCS	Right AT	LAC	2	–
11	M	28	15	GTCS	Right AT and MT	LAC + VPA	15	–
12	F	28	6	FBTCS	Right AT and MT	OXC	4	–
13	F	28	3	FBTCS	Right AT and MT	OXC	1	–
14	F	28	10	GTCS	Right AT	OXC + VPA	7	rHS
15	M	25	18	FBTCS	Right AT and MT	OXC	16	rHS
16	M	22	19	GTCS	Right AT and MT	CBZ + VPA	18	rHS
17	M	39	20	FBTCS	Right AT	CBZ	18	–
18	F	23	3	FBTCS	Right AT	CBZ	1	–
19	F	29	5	FBTCS	Right AT	LTG	2	rHS
20	F	28	6	FBTCS	Right AT and MT	OXC	4	rHS
21	M	31	20	FBTCS	Right AT and MT	LTG + VPA	17	–
22	F	15	4	GTCS	Right AT and MT	OXC + LTG	2	–
23	F	29	9	GTCS	Right MT	LTG + VPA	8	–
24	F	36	4	FBTCS	Right AT	LAC	3	–
25	F	28	9	FBTCS	Right MT	TPM	7	–
26	F	43	21	GTCS	Right AT and MT	CBZ + VPA	21	rHS
27	M	36	11	GTCS	Right AT and MT	LTG + VPA + CBZ	11	–
28	M	44	6	FBTCS	Right AT and MT	LTG	5	–
29	M	43	15	GTCS	Right AT	LTG + VPA + LEV	14	–
30	F	40	18	GTCS	Right AT	LTG + CBZ	17	–

FBTCS, focal to bilateral tonic–clonic seizures; GTCS, generalized tonic–clonic seizures; AT, anterior temporal; MT, middle temporal; ASM, antiepileptic drugs; LTG, lamotrigine; LEV, levetiracetam; OXC, oxcarbazepine; VPA, sodium valproate; CBZ, carbamazepine; LAC, lacosamide; TPM, topiramate; rHS, right hippocampal sclerosis.

### 3.2 Modularity Q of the FC network

**Figure 1:**
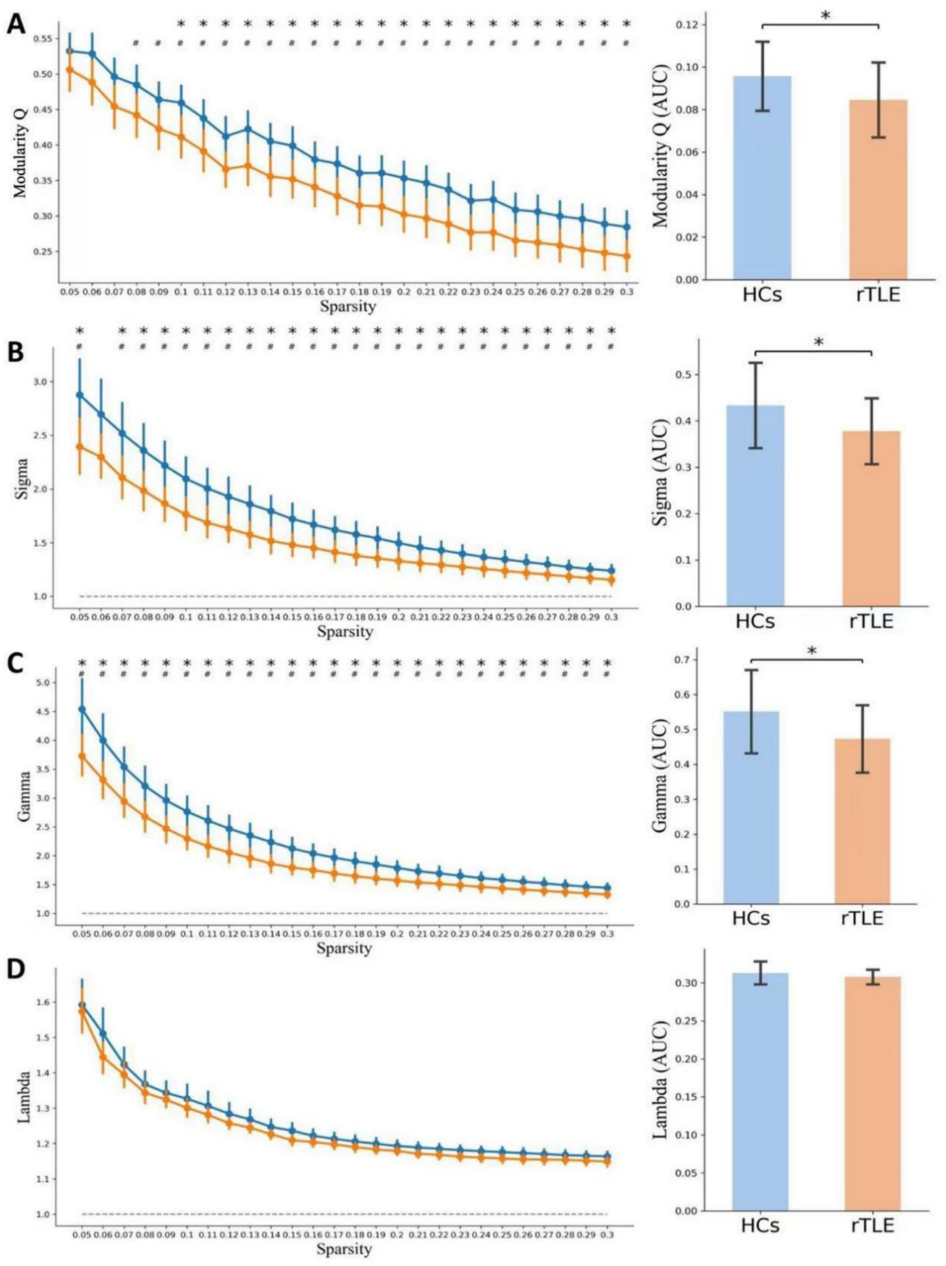
Global topological properties in the two groups. The global topological properties of healthy controls (HCs, blue) and right temporal lobe epilepsy (rTLE) patients (orange) are displayed. The left column presents topological metrics at each network sparsity (0.05–0.3, step = 0.01), with vertical lines representing the mean and 95% confidence intervals obtained from 1,000 bootstrap iterations. The right column shows the area under the curve (AUC) of topological properties across the sparsity range. **(A)** Q, **(B)** Sigma, *σ*, **(C)** Gamma, *γ*, **(D)** Lambda, *λ*. # indicates *p* < 0.05 (uncorrected), compared at each network sparsity level. * in the left column indicates *p* < 0.05 (FDR corrected) across all sparsity levels. * in the right column indicates *p* < 0.05 (comparison of AUC for each global topological property between groups).

Compared with HCs, the AUC of modularity Q across sparsity levels ranging from 5 to 30% (AUC_HCs_ = 0.0955 ± 0.0164 [mean ± SD], AUC_rTLE_ = 0.0841 ± 0.0179, *p* = 0.0129) and the modularity Q at 15% sparsity (Q_HCs_ = 0.3884 ± 0.0738 [mean ± SD], Q_rTLE_ = 0.3333 ± 0.0704, *p* = 0.0044) were both significantly decreased in rTLE patients (Figure 1A).

### 3.3 Modular partitions and interactions of the FC network

Using the community partition algorithm described above, we found that the community partitions were highly consistent under sparsity levels ranging from 0.05 to 0.30 (step size 0.01). For more detailed analysis, community partitions were performed at 15% sparsity in both groups, a threshold commonly used in previous studies, allowing us to capture the underlying modular organization while maintaining network connectedness.

**Figure 2:**
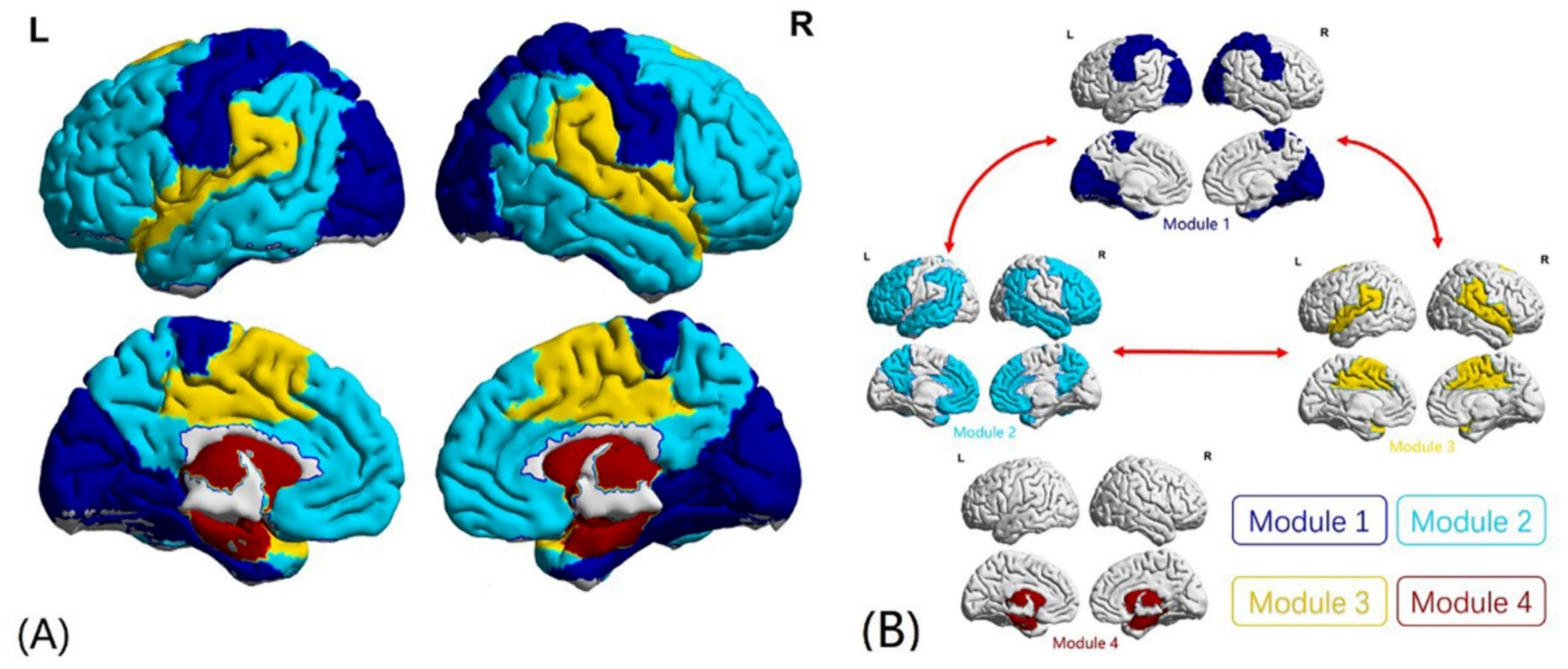
Modular partitions of the functional connectivity (FC) network and modular interaction. **(A)** Modular partitions of the FC network at 15% sparsity. Modules 1, 2, and 3 were primarily located in the cortex, while module 4 was predominantly located in subcortical areas. Different colors represent distinct network modules. **(B)** Comparison of adjusted strength of modular interaction (ASMI) between the two groups. Compared to healthy controls (HCs), interactions between modules 1, 2, and 3 were significantly enhanced in right temporal lobe epilepsy (rTLE) patients, as indicated by red arrows. No significant differences were observed between module pairs 1–4, 2–4, and 3–4.

The partition results showed that the whole brain FC network at the overall group level could be divided into four modules (Figure 2A, Table 4). In this study, the modular interaction was quantified by ASMI value as mentioned before. Compared with the HCs, the ASMI values of modules 1–2, 1–3, and 2–3 in the rTLE group were increased, while the ASMI values of modules 1–4, 2–4, and 3–4 did not differ significantly different between the two groups (Figure 2B, Table 5).

**Table 4:** The community partitions (4 modules).

Module 1	Module 2		Module 3	Module 4
Precentral_L	Frontal_Sup_L	Cingulum_Ant_L	Rolandic_Oper_L	Hippocampus_L
Precentral_R	Frontal_Sup_R	Cingulum_Ant_R	Rolandic_Oper_R	Hippocampus_R
Calcarine_L	Frontal_Sup_Orb_L	Cingulum_Post_L	Supp_Motor_Area_L	ParaHippocampal_L
Calcarine_R	Frontal_Sup_Orb_R	Cingulum_Post_R	Supp_Motor_Area_R	ParaHippocampal_R
Cuneus_L	Frontal_Mid_L	Parietal_Inf_L	Insula_L	Amygdala_L
Cuneus_R	Frontal_Mid_R	Parietal_Inf_R	Insula_R	Amygdala_R
Lingual_L	Frontal_Mid_Orb_L	Angular_L	Cingulum_Mid_L	Caudate_L
Lingual_R	Frontal_Mid_Orb_R	Angular_R	Cingulum_Mid_R	Caudate_R
Occipital_Sup_L	Frontal_Inf_Oper_L	Precuneus_L	SupraMarginal_L	Thalamus_L
Occipital_Sup_R	Frontal_Inf_Oper_R	Precuneus_R	SupraMarginal_R	Thalamus_R
Occipital_Mid_L	Frontal_Inf_Tri_L	Temporal_Mid_L	Putamen_L	
Occipital_Mid_R	Frontal_Inf_Tri_R	Temporal_Mid_R	Putamen_R	
Occipital_Inf_L	Frontal_Inf_Orb_L	Temporal_Pole_Mid_L	Pallidum_L	
Occipital_Inf_R	Frontal_Inf_Orb_R	Temporal_Pole_Mid_R	Pallidum_R	
Fusiform_L	Olfactory_L	Temporal_Inf_L	Heschl_L	
Fusiform_R	Olfactory_R	Temporal_Inf_R	Heschl_R	
Postcentral_L	Frontal_Sup_Medial_L		Temporal_Sup_L	
Postcentral_R	Frontal_Sup_Medial_R		Temporal_Sup_R	
Parietal_Sup_L	Frontal_Med_Orb_L		Temporal_Pole_Sup_L	
Parietal_Sup_R	Frontal_Med_Orb_R		Temporal_Pole_Sup_R	
Paracentral_Lobule_L	Rectus_L			
Paracentral_Lobule_R	Rectus_R			

**Table 5:** Intermodular average strength of modular interaction (ASMI) value of the two groups.

Modular interaction	rTLE (mean ± SD)	HC (mean ± SD)	*p*-value	FDR adjusted *p*-value
Module 1–2	0.7591 ± 0.1958	0.6574 ± 0.1249	0.0190	0.0380*
Module 1–3	0.7751 ± 0.2036	0.6653 ± 0.1369	0.0180	0.0380*
Module 1–4	0.6719 ± 0.2776	0.5460 ± 0.2129	0.0520	0.0780
Module 2–3	0.7637 ± 0.2049	0.6534 ± 0.1224	0.0134	0.0380*
Module 2–4	0.6660 ± 0.2668	0.6355 ± 0.1337	0.5901	0.5901
Module 3–4	0.7060 ± 0.2356	0.6120 ± 0.1669	0.0778	0.0934

*Indicates that passed the FDR correction.

### 3.4 Measurement of CH number and PC/WMD values of FC networks

There were no statistical differences in the total number of CHs/non-CH nodes in all HC subjects’ FC networks, and that of rTLE patients’ FC networks (HC vs. rTLE: 332/2368[30 × 90–332] vs. 371/2,329[30 × 90–371], Chi-square test, *p* = 0.1148). In addition, the average PC/WMD value of all CHs in the HC group was significantly higher than that of the rTLE group (HC vs. rTLE: 0.3655 ± 0.0425 vs. 0.3942 ± 0.0427 [mean ± SD], two-sample *t*-test, two-tailed, *p* = 0.0114).

### 3.5 Network-based statistics analysis of major CHs’ FC networks

**Figure 3:**
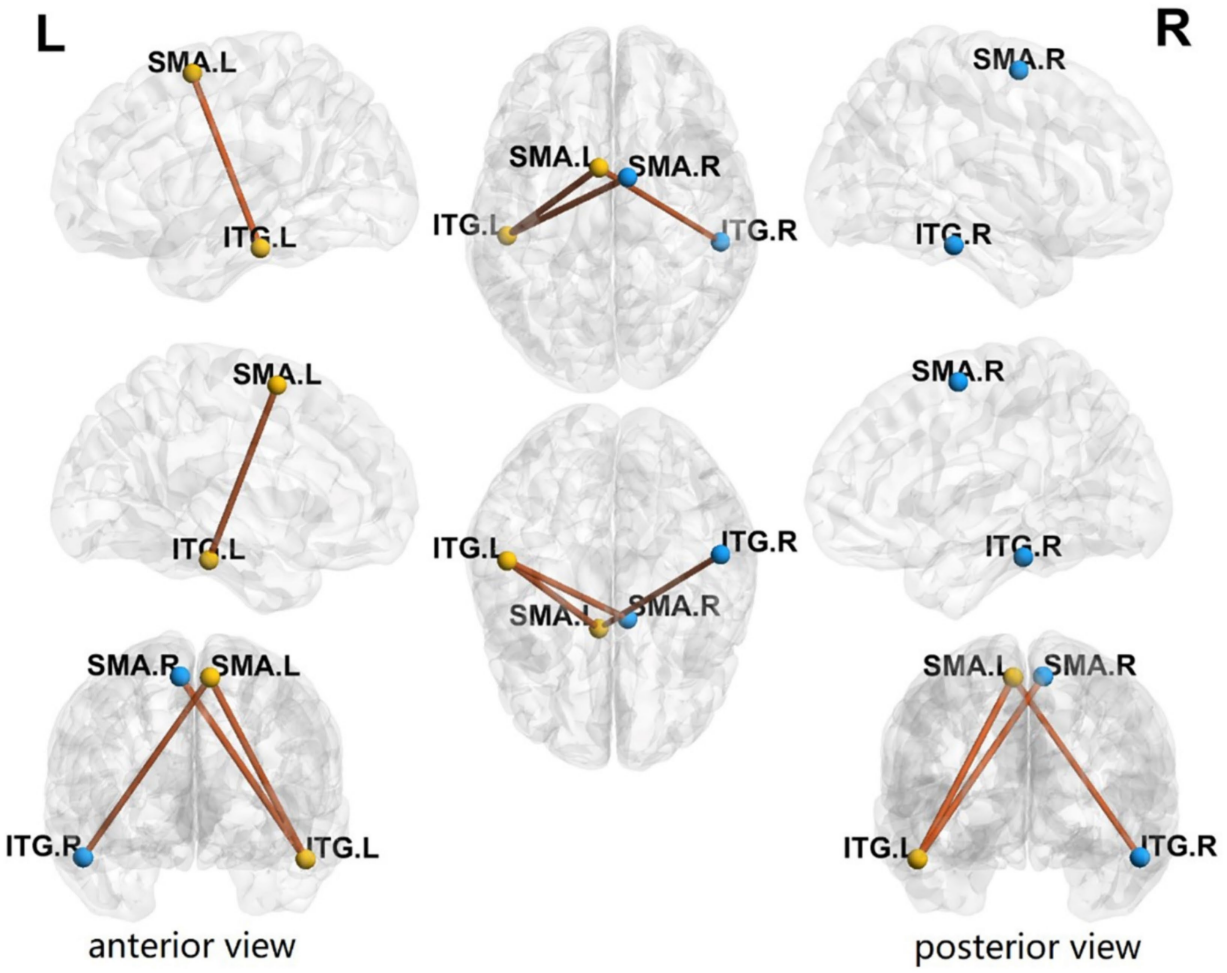
Comparison of major connector hubs’ (CHs) functional connectivity networks by NBS. Plots of the comparison of major connector hubs’ (CHs) functional connectivity (FC) networks between healthy controls (HCs) and right temporal lobe epilepsy (rTLE) patients, analyzed using network-based statistics (NBS). Compared to HCs, rTLE patients exhibited enhanced FC strength in the network components formed by the left and right supplementary motor areas (SMA.L, SMA.R) and the left and right inferior temporal gyri (ITG.L, ITG.R). The connections between these regions are depicted as orange lines, with different perspectives provided: anterior and posterior views.

Compared with the HC group, a network component composed of 4 CHs the left and right inferior temporal gyrus (ITG.L and ITG.R) and the left and right supplementary motor areas (SMA.L and SMA.R), and 3 connections between them showed significantly enhanced FC strength in rTLE patients (*p* = 0.017, Figure 3).

### 3.6 Topological properties of FC networks and CHs

**Figure 4:**
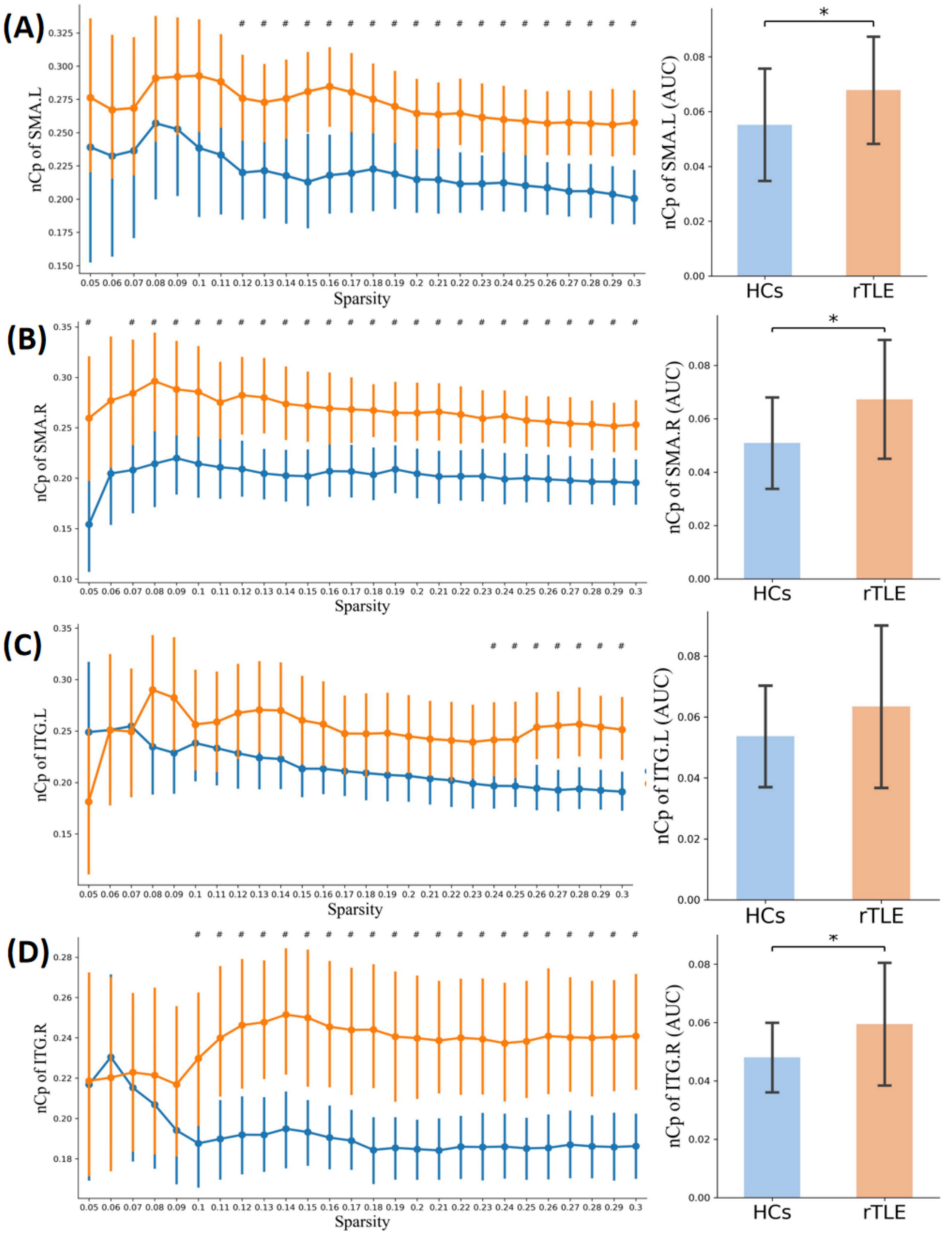
Nodal clustering coefficient in the two groups. Plots of the nCp in key connector hubs, comparing HCs (blue) and rTLE patients (orange). The left column presents nCp values across network sparsity levels (0.05–0.30, step = 0.01), with vertical lines indicating the mean and 95% confidence intervals, derived from 1,000 bootstrap iterations. The right column illustrates the area under the curve (AUC) for each region. **(A)** Left supplementary motor area (SMA.L), **(B)** Right supplementary motor area (SMA.R), **(C)** Left inferior temporal gyrus (ITG.L), **(D)** Right inferior temporal gyrus (ITG.R). # indicates *p* < 0.05 (uncorrected), comparison at each network sparsity level. * indicates *p* < 0.05 (FDR-corrected), comparison of AUC values across the four connector hubs.

**Figure 5:**
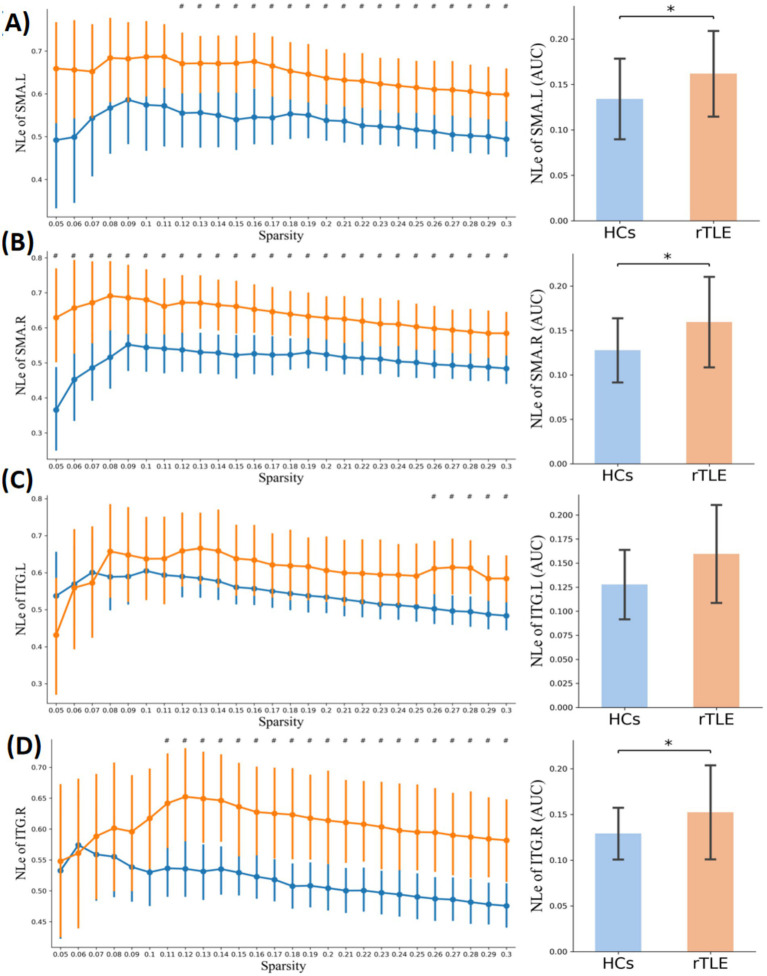
Nodal local efficiency in the two groups. Plots of nodal local efficiency (NLE) in key connector hubs, comparing healthy controls (HCs, blue) and right temporal lobe epilepsy (rTLE) patients (orange). The left column displays NLE values across network sparsity levels (0.05–0.30, step = 0.01), with vertical lines indicating the mean and 95% confidence intervals, derived from 1,000 bootstrap iterations. The right column illustrates the area under the curve (AUC) for each region. **(A)** Left supplementary motor area (SMA.L), **(B)** Right supplementary motor area (SMA.R), **(C)** Left inferior temporal gyrus (ITG.L), **(D)** Right inferior temporal gyrus (ITG.R). # indicates *p* < 0.05 (uncorrected), comparison at each network sparsity level. * indicates *p* < 0.05 (FDR-corrected), comparison of AUC values across the four connector hubs.

**Figure 6:**
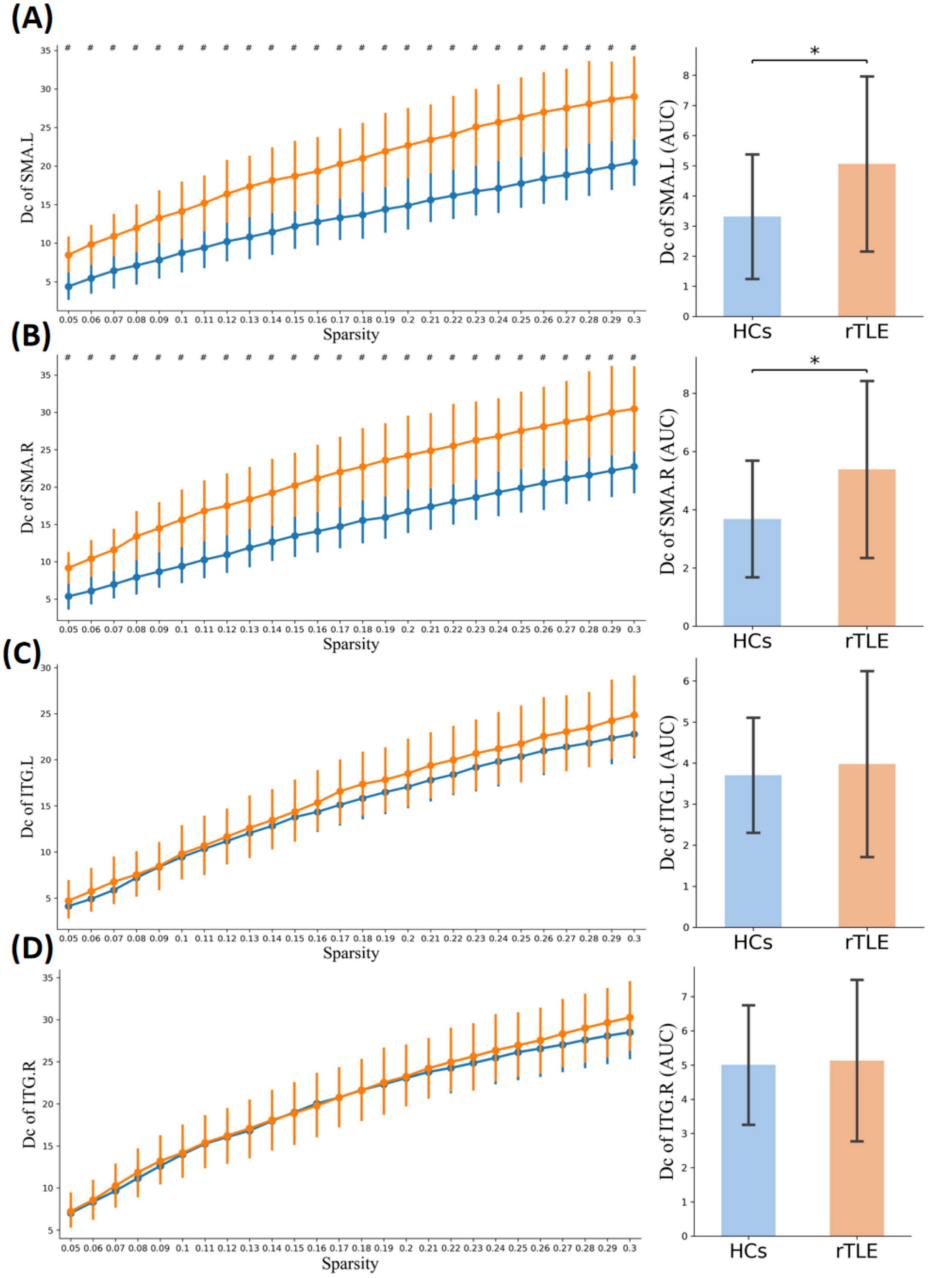
Nodal degree centrality in the two groups. Plots of nodal degree centrality (Dc) in key connector hubs, comparing healthy controls (HCs, blue) and right temporal lobe epilepsy (rTLE) patients (orange). The left column displays Dc values across network sparsity levels (0.05–0.3, step = 0.01), with vertical lines indicating the mean and 95% confidence intervals, derived from 1,000 bootstrap iterations. The right column illustrates the area under the curve (AUC) for each region. **(A)** Left supplementary motor area (SMA.L), **(B)** Right supplementary motor area (SMA.R), **(C)** Left inferior temporal gyrus (ITG.L), **(D)** Right inferior temporal gyrus (ITG.R). # indicates *p* < 0.05 (uncorrected), comparison at each network sparsity level. * indicates *p* < 0.05 (FDR corrected), comparison of AUC values across the four connector hubs.

Within the sparsity range of 0.05 to 0.30, the AUC of the small-world index (*σ*) and the normalized clustering coefficient (*γ*) of the rTLE FC networks were decreased compared with the HC FC networks, the AUC of node clustering coefficient and node local efficiency of SMA.L, SMA.R, and ITG.R were increased, and the AUC of node degree centrality of SMA.L, SMR.R were increased as well (Table 6, Figures 1B–D, 4–6).

**Table 6:** The AUC of topological properties in the two groups (0.05–0.30 sparsity range).

Topological properties	CH (node)	rTLE (mean ± SD)	HC (mean ± SD)	p-value	FDR adjusted p-value
Sigma (σ)	–	0.3778 ± 0.0722	0.4332 ± 0.0935	0.0062	–
Gamma (γ)	–	0.4731 ± 0.0979	0.5512 ± 0.1216	0.0081	
Lambda (λ)	–	0.3077 ± 0.0098	0.3131 ± 0.0154	0.1196^#^	
Node clustering coefficient	SMA.L	0.0678 ± 0.0199	0.0552 ± 0.0208	0.0198	**0.0264** ^*^
	SMA.R	0.0673 ± 0.0227	0.0509 ± 0.0174	0.0027	**0.0108** ^*^
	ITG.L	0.0634 ± 0.0271	0.0537 ± 0.0169	0.1034^#^	0.1034
	ITG.R	0.0595 ± 0.0214	0.0480 ± 0.0121	0.0130^#^	**0.0260** ^*^
Node local efficiency	SMA.L	0.1619 ± 0.0479	0.1341 ± 0.0452	0.0245	**0.0435** ^*^
	SMA.R	0.1596 ± 0.0518	0.1277 ± 0.0367	0.0080	**0.0320** ^*^
	ITG.L	0.1538 ± 0.0642	0.1367 ± 0.0307	0.1986^#^	0.1986
	ITG.R	0.1523 ± 0.0522	0.1291 ± 0.0288	0.0326^#^	**0.0435** ^*^
Node degree centrality	SMA.L	5.0586 ± 2.9554	3.3109 ± 2.1030	0.0102^#^	**0.0256** ^*^
	SMA.R	5.3848 ± 3.0906	3.6811 ± 2.0393	0.0128^#^	**0.0256** ^*^
	ITG.L	3.9786 ± 2.3022	3.7031 ± 1.4264	0.5627^#^	0.7503
	ITG.R	5.1303 ± 2.4028	5.0022 ± 1.7744	0.8150	0.8150

^#^Indicates that passed permutation test; *Indicates that passed the FDR correction.

### 3.7 Altered interhemispheric structural connectivity

**Figure 7:**
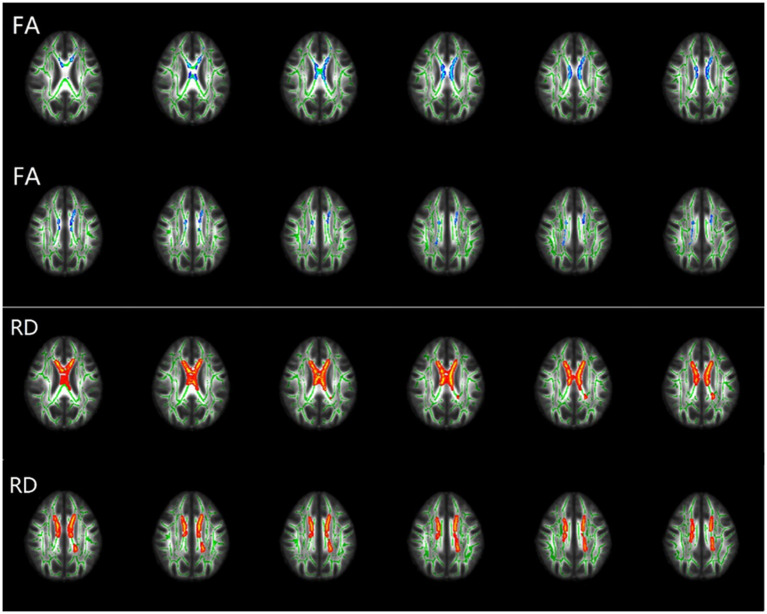
TBSS results of FA and RD differences between HCs and rTLE patients. Plots of tract-based spatial statistics (TBSS) results, comparing fractional anisotropy (FA) and radial diffusivity (RD) between healthy controls (HCs) and right temporal lobe epilepsy (rTLE) patients. Green represents the mean FA skeleton of all participants. Blue regions indicate the areas with significantly decreased FA in rTLE patients (first and second rows). Red regions indicate the areas with significantly increased RD in rTLE patients (third and fourth rows).

Compared to the HC group, the TBSS analyses revealed significantly decreased FA and increased RD of the corpus callosum in the rTLE group, while the AD and MD was not statistically different between groups (Figure 7).

### 3.8 Cortical morphologic CH changes

**Figure 8:**
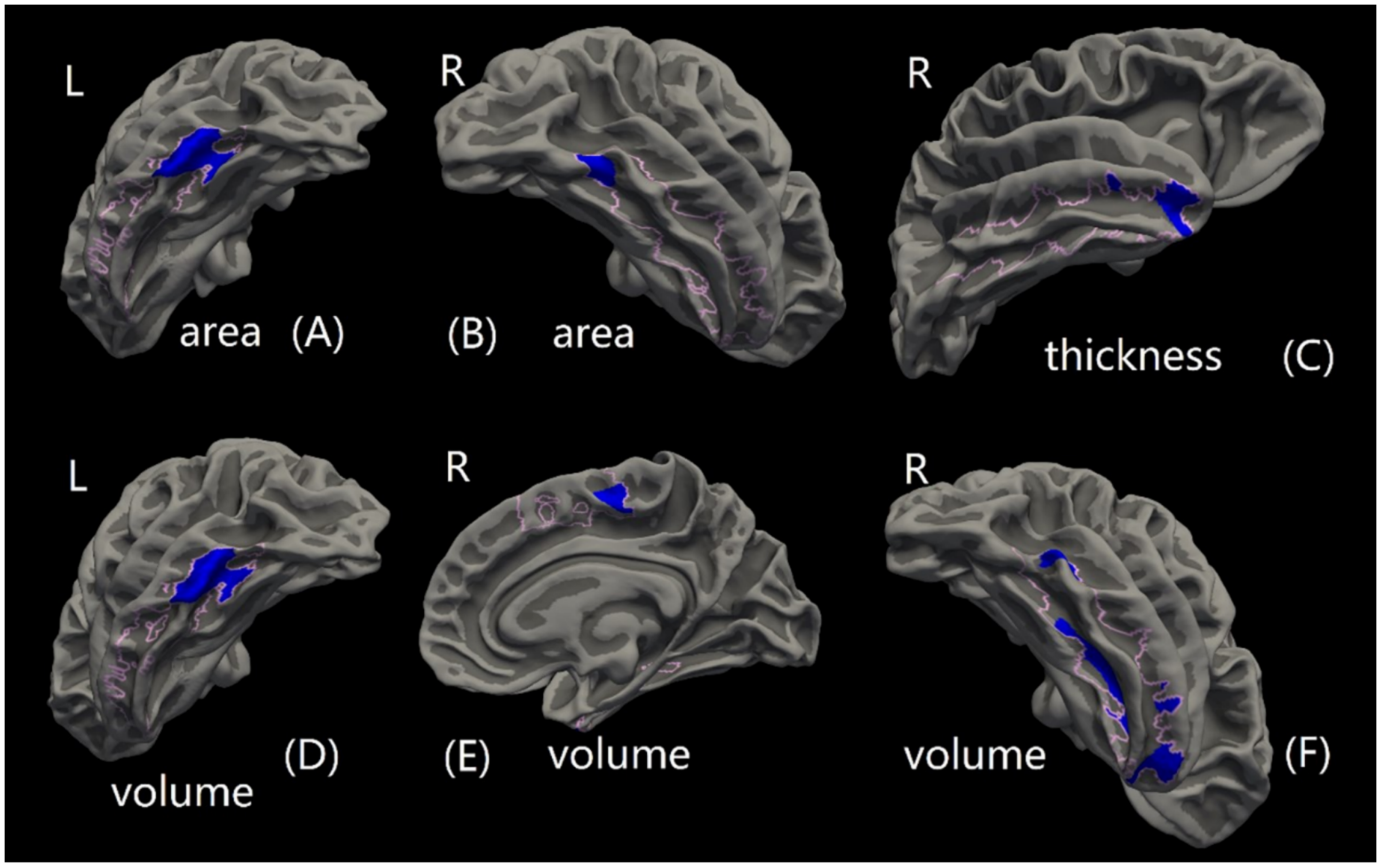
Cortical morphological changes of connector hubs (CHs). Plots of cortical morphological alterations in right temporal lobe epilepsy (rTLE) patients compared to healthy controls (HCs). Pink boundaries indicate the surface areas where the three regions of interest (ROIs)—right inferior temporal gyrus (ITG.R), left inferior temporal gyrus (ITG.L), and right supplementary motor area (SMA.R)—from the AAL90 template were mapped from the MNI152 standard space to the FreeSurfer fsaverage space. Blue clusters represent cortical regions with significantly reduced anatomical structure metrics in rTLE patients compared to HCs. **(A)** Cluster of decreased cortical surface area in the ITG.L region. **(B)** Cluster of decreased cortical surface area in the ITG.R region. **(C)** Clusters of decreased cortical thickness in the ITG.R region. **(D)** Clusters of decreased cortical volume in the ITG.L region. **(E)** Cluster of decreased cortical volume in the SMA.R region. **(F)** Clusters of decreased cortical volume in the ITG.R region.

Compared to the HC group, the rTLE group had 1 cluster that showed decreased cortical surface area and 3 clusters that showed reduced cortical volume in the ITG.L region, 1 cluster showing cortical volume decreases in the SMA.R region, 1 decreased cortical surface area cluster, 5 decreased cortical volume clusters, and 2 decreased cortical thickness clusters in the ITG.R region. No significant differences between group were found in SMA.L cortical surface area, volume, and thickness, ITG.L cortical thickness, or SMA.R cortical surface area and thickness (Figure 8, Table 7).

**Table 7:** Significant clusters of cortical morphologic CH changes between the two groups.

Cortical anatomical metrics	CH (In AAL90 template)	Significant cluster	Cluster area (mm^2^)	Cluster vertex	Cluster *p*-value	Cluster location^a^
Thickness	ITG.R	1	232.41	358	0.0004	Inferior temporal
		2	38.63	56	0.0012	Middle temporal
Surface area	ITG.L	1	804.98	1,254	0.0004	Inferior temporal
	ITG.R	1	235.9	366	0.0004	Fusiform
Volume	ITG.L	1	385.88	624	0.0004	Inferior temporal
		2	110.39	157	0.0004	Inferior temporal
		3	24.25	44	0.0004	Inferior temporal
	SMA.R	1	164.22	302	0.0004	Paracentral
	ITG.R	1	330.32	700	0.0004	Fusiform
		2	250.48	390	0.0004	Inferior temporal
		3	191.9	262	0.0004	Inferior temporal
		4	45.68	68	0.0004	Middle temporal
		5	9.81	14	0.0012	Middle temporal

The numbers in this table refer to the reduced values of the rTLE group compared to the HC group.

a Represents the cluster anatomical location in the FreeSurfer Desikan-Killiany partition template.

## 4 Discussion

In this study, using multimodal neuroimaging techniques and network analysis methods, we investigated the changes in modular interactions and CH topological alterations in rTLE patients’ functional brain networks and explored the corresponding anatomical changes. The significant findings were as follows: (1) The small-world index, normalized clustering coefficient and modularity Q were decreased in rTLE patient functional brain networks. (2) Interactions between modules 1, 2, and 3 were enhanced in rTLE patients. (3) The average PC/WMD value of all CHs in the rTLE FC network was increased. (4) In the FC network composed of only major CHs, a network component composed of 4 CHs (ITG.L, ITG.R, SMA.L, and SMA.R) showed significantly enhanced FC strength in rTLE patients. (5) The node-level topological properties findings revealed that rTLE patients had significantly increased node clustering coefficient and node local efficiency in the SMA.L, SMA.R, and ITG.R, and increased node degree centrality in the SMA.L and SMR.R. (6) TBSS analysis showed decreased FA and increased RD of the corpus callosum of rTLE patients. (7) Finally, several cortical anatomical metrics were decreased in the ITG.R, ITG.L, and SMA.R of rTLE patients.

Modularity has been widely regarded as one of the main organization principles in most complex biological networks, with most studies focusing on modular division, reconfiguration, specific modular functions, or integration and segregation (38, 40–42). Moreover, research on the interaction changes between functional modules in rTLE patients and how it affects clinical manifestations is even rarer. Our research began with the modular structure of functional brain networks and uncovered changes in the modular interaction of rTLE patients by constructing an overall level modular template, and the differences in global FC network topological properties, which are represented by small-world attributes, were also compared. It has been proven that the ability of human networks to organize with optimal efficiency is due to the small-world topology allowing simultaneous processing of global and local parallel information (43, 44). The previous study on idiopathic generalized epilepsy has shown decreased small-world index both in functional and structural connectivity networks, and suggested that a less optimized network organization caused by the specific pathological state occurred in idiopathic generalized epilepsy patients (45). Our findings demonstrated that the rTLE patients presented a decreased small-world index, which suggests that they may also have a similar network pathological state. Moreover, the normalized clustering coefficient (*γ*) (46), the decreased γ in rTLE patients suggests that the increased randomness in the large-scale functional brain network of rTLE may underlie an increased likelihood of generalization of epileptic discharges, but still need further exploration.

Modularity is an important metric to measure network efficacy and brain health (47, 48). Modularity Q value is associated with network segregation and integration, describing the extent to which a network can be divided into non-overlapping groups with high within-module connections and low between-module connections. Decreased modularity in the functional brain network was reported in TLE patients, and the late onset mesial TLE patients showed the largest reduction than the early onset (49). Our results also showed decreased modularity in the rTLE FC network, which reflected a less organized network of cohesive subdivisions. However, few studies have further explored the changes in the modular interaction in rTLE patients after modular structure is damaged. Physics studies of the spreading dynamics in hierarchical cluster networks have shown that sparse connections between modules limit global spreading and tight connections within modules sustain recurrent local activity, but few have further studied FC networks in TLE to see whether FC modular structure damage would facilitate modular interaction. To address this problem, we analyzed the characteristics of module partitions and the ASMI in FC networks. The division of modules in this study showed that modules 1, 2, and 3 were mostly located in the cortex, and module 4 was mostly located in subcortical areas. Meanwhile, interactions between modules 1, 2, and 3 were enhanced in the rTLE patients. These suggests strengthened functional coupling between the cortical modules of rTLE patients, but whether it is related to the epileptic electrical signals spread across the entire cerebral cortex remains to be further investigated.

In brain networks, information transmission relies critically on a limited set of internal key nodes known as CHs. Although few in number, these CHs facilitate a disproportionately large share of overall neural communication (8). We therefore hypothesized that the broad alterations in functional connectivity and enhanced modular interactions observed in patients with rTLE may stem from changes in the number and/or topological properties of CHs. Previous studies have reported that an overall increase in PC and a decrease WMD in cortical networks are associated with susceptibility to generalized seizures in TLE patients (6). Consistent with this, our findings revealed that the average PC and WMD values across all CHs were significantly altered in rTLE patients compared to controls, although the total number of CHs remained unchanged. These results suggest that the enhanced modular interaction in rTLE is not attributable to a change in the quantity of CHs, but rather to topological reorganization specifically, changes in PC and WMD values. This indicates that CHs in rTLE patients exhibit a stronger tendency to communicate with nodes outside their own module rather than within it.

TLE has attracted much attention because of its frequent partial seizures followed by generalized seizures. Altered functional connectivity in the hemisphere contralateral to the epileptic zone is often seen in TLE patients, regardless of lateralization (50, 51). Our results also showed contralateral enhanced SMA.L-ITG.L FC in rTLE patients, which is consistent with these studies. However, few studies had focused on the functional connectivity of CHs across hemispheres. By performing an NBS analysis, we found that an enhanced FC network component spanned both hemispheres in the rTLE group. However, whether this change is related to the spread of interhemispheric epileptic foci discharge remains unclear and needs to be further explored. Further analysis of CHs in this network component revealed that they were located at symmetrical positions in the bilateral hemispheres, and that SMA.L-ITG.R and SMA.R-ITG.L FC were both enhanced. Moreover, both SMA.L and SMA.R showed increased node clustering coefficient, node local efficiency, and degree centrality. This suggests that the SMA as a CH is not only closely functionally connected to neighboring areas in rTLE patients, but also plays an important mediating role in the enhanced FC across hemispheres. A previous combined EEG-fMRI study on juvenile myoclonic epilepsy indicated that positive blood oxygenation level-dependent responses were highly correlated with the high EEG-network variation in the SMA region, which was related to epileptic dynamic network organization (52). The increased node clustering coefficient and local efficiency of ITG.R also indicated tight functional connections with its neighboring areas. This suggests that the ITG.R and SMA in rTLE patients may play an important role in the enhanced major CHs’ FC network and interhemispheric functional connectivity.

The formation of brain FC is based on anatomical structure, and abnormal FC is often accompanied by structural alterations, including structural connection and local anatomical morphology, which has been shown by previous studies (13, 53, 54). However, there are still few studies focusing on the structural alterations corresponding to FC between modular interactions and CHs in TLE. Therefore, we explored the structural changes in rTLE patients by studying the whole brain white matter fiber skeleton and the local anatomical structure of CHs. The decreased FA and increased RD in corpus callosum suggest some orientation-dependent aspects of the microstructure of rTLE were changed and the structural connections between bilateral cerebral hemispheres might also change, but whether the enhanced FC between interhemispheric CHs is an adaptive mechanism to compensate for altered interhemispheric structural connectivity remains to be further studied. Finally, we explored the local cortical anatomical structure changes of CHs in the enhanced FC network component of rTLE patients. The most obvious change in the cortical structure was seen in the ITG.R, where volume decrease was the most prominent feature. This suggested that the ITG.R, as part of the right temporal lobe which is closely related to rTLE, not only plays a major role in the enhanced FC network of CHs but also exhibits large anatomical change. The interaction between altered FC and structure eventually leads to the recurrent onset of symptoms and disease progression, reduced neurite density in ipsilateral fusiform gyri of basal temporal regions has been seen in a microstructural imaging study of TLE patients (55), and pathological research also indicated neuronal loss and gliosis in this area. A previous study on TLE patients with depression showed decreased cortical volume of the SMA.R, implying possible impaired explicit emotion regulation (56). This morphological change was consistent with our results, but whether it is related to the abnormal mental status of rTLE patients still needs further study. In addition, we also found decreased ITG.L cortical volume and surface area, indicating that rTLE not only affects the functional topological properties of CHs within the enhanced FC network component, but also their underlying anatomical structure. Previous studies also reported reduced cortical thickness in TLE (21, 57–59), suggesting a widespread changed topographic distribution of cortical morphology. However, there are few studies explaining this observation from a structural or functional network approach, and whether ipsilateral cortical structure changes in rTLE affect the contralateral cortical structure remains unclear and needs further exploration.

Although our study conducted a detailed functional analysis of the topological properties and corresponding structural changes from the overall network level to local CHs in rTLE patients, there were still some limitations. First, limited by the sample size, clinical characteristics, and confounding effects of anti-epileptic drugs, we were unable to perform further subgroup analysis according to seizure type and specific drugs taken. MRI studies also showed differential functional connectivity and brain morphology between subjects with left and right TLE (60, 61), and it is necessary to have a cohort study of rTLE and left TLE in the future for more in-depth analysis. In addition, the rs-fMRI sequence in this study has a larger slice thickness, and acquiring higher resolution data would allow us to make stronger inferences in our further studies. Another limitation is the small sample size, which reduces the statistical power of the analyses and does not allow for adequate stratification by clinical variables such as seizure type. Moreover, to increase the sample size, we included seven patients with 5-mm slice thickness. Furthermore, while this study utilized the well-established ILAE classification framework for patient inclusion, we acknowledge that clinical diagnostic data can be inherently affected by uncertainties and imprecisions. These challenges in data interpretation highlight a potential avenue for future methodological refinement. Recently, computational tools based on fuzzy logic, specifically designed to operate within ambiguous environments, have been recommended for such tasks. For instance, fuzzy similarity approaches, as demonstrated by Versaci et al. in a different context, offer a powerful framework for handling uncertainty by grouping data in a fuzzy sense and extracting discriminative features from each group. This not only facilitates robust classification but also achieves this with reduced computational complexity. Although not implemented here, the adoption of such fuzzy logic-based tools could be highly valuable in future epilepsy research to enhance the precision and reliability of classification models that must contend with imperfect or vague data. Although the rs-fMRI data of all participants were using the same standard preprocessing procedures to minimize the influence on the results, further studies with larger samples are needed for validating the present findings. Second, our study lacked dynamic FC analysis. Previous studies have shown that by adopting dynamic functional network topological properties analyses, more accurate results can be achieved in identifying laterality in TLE patients compared with static FC analysis (62). Further exploration of this aspect can provide more powerful theoretical support for the modular information exchange, and the amount and direction of information flowing in CHs. Third, due to the limitations of the current brain imaging and network analysis methods, the use of different brain partition templates or network metric algorithms may lead to different experimental results (63), and there is still controversy about whether voxel-based morphometry (VBM) or surface-based morphometry (SBM) is better in image data processing (64, 65). We now explicitly acknowledge that we did not exhaustively evaluate alternative *τ* (agreement-matrix threshold) or the Louvain resolution parameter, and we outline this as a priority for future work with larger samples and computational resources. We also indicate that our code and analysis steps are organized to enable such sensitivity analyses in follow-up studies. Moreover, the fiber connections between the 4 CHs (SMA.L, SMA.R, ITG.L, ITG.R) may be long-distance and complex. Therefore, we used TBSS for the whole-brain white matter approach rather than tracking fiber connections between these regions to obtain better sensitivity and objectivity results. However, employing whole-brain white matter tracking may not be very specific to the fiber connections between these CHs and future studies are needed as the methodology improves.

## 5 Conclusion

In this study, we integrated multimodal MRI with graph theory methodologies to systematically investigate alterations in functional network modular interactions and the topological properties of core hubs (CHs) in patients diagnosed with right temporal lobe epilepsy (rTLE), while simultaneously exploring the structural modifications that are associated with these functional and topological changes. Our findings revealed that the global properties characterized by small-world attributes in rTLE brain functional networks were altered, with a decrease in modularity degree. The functional networks exhibited a tendency towards generalization, potentially due to enhanced modular interactions, primarily attributed to an increased PC/WMD ratio of CHs. Specifically, the functional connectivity (FC) network component comprising four CHs (left supplementary motor area [SMA.L], right supplementary motor area [SMA.R], left inferior temporal gyrus [ITG.L], and right inferior temporal gyrus [ITG.R]) was significantly enhanced in the rTLE patients, and the topological properties of these CHs were also modified. Furthermore, corresponding structural changes included altered fractional anisotropy (FA) and radial diffusivity (RD) in the corpus callosum, as well as local cortical structure modifications in SMA.R, ITG.L, and ITG.R. Collectively, our study offers novel insights into the functional and structural mechanisms underlying rTLE.

## Data Availability

The raw data supporting the conclusions of this article will be made available by the authors, without undue reservation.

## Footnotes

1
https://rfmri.org/DPABI

2
https://sites.google.com/site/bctnet/

3
http://www.nitrc.org/projects/gretna

4
http://www.nitrc.org/projects/nbs/

5
http://www.nitrc.org/projects/panda

6
https://fsl.fmrib.ox.ac.uk/fsl/fslwiki/

7
http://surfer.nmr.harvard.edu/
